# Histological and biochemical evaluation of plasma rich in growth factors treatment for grade II muscle injuries in sheep

**DOI:** 10.1186/s12917-022-03491-2

**Published:** 2022-11-12

**Authors:** Daniel Aguilar-García, J. Andrés Fernández-Sarmiento, María del Mar Granados Machuca, Juan Morgaz Rodríguez, Pilar Muñoz Rascón, Rocío Navarrete Calvo, Yolanda Millán Ruiz, José María Carrillo Poveda, Juan Muñoz Castañeda, Ramón Cugat Bertomeu, Juan Manuel Domínguez Pérez

**Affiliations:** 1 Departamento de Medicina y Cirugía Animal, Universidad de Córdoba. Campus Universitario de Rabanales, 14014 Córdoba, Spain; 2 Fundación García-Cugat para Investigación Biomédica, Plaza Alfonso Comín 5-7, 08023 Barcelona, Spain; 3 Departamento de Anatomía y Anatomía Patológica Comparadas, Universidad de Córdoba, Campus Universitario de Rabanales, 14014 Córdoba, Spain; 4 Departamento de Medicina y Cirugía Animal, Cátedra García Cugat, Universidad CEU Cardenal Herrera, 46115 Valencia, Spain; 5 Instituto Maimónides de Investigación Biomédica de Córdoba, Universidad de Córdoba, 14014 Córdoba, Spain; 6 Instituto Cugat, Hospital Quirón Salud, Plaza Alfonso Comín 5-7, 08023 Barcelona, Spain

**Keywords:** Muscle injury, Platelet-rich plasma, Plasma rich in growth factors, Muscle repair

## Abstract

The purpose of this study was to perform a histological and biochemical evaluation of the influence of plasma rich in growth factors (PRGF) on muscle regeneration process after a surgically induced grade II muscle laceration. A randomized, single blind, controlled experimental research was conducted including twenty-one adult healthy sheep, randomly divided in three groups (n = 7). A grade II surgical section was performed in the biceps femoris muscle of both hindlimbs. After two days (basal time), intralesional infiltration of autologous PRGF or Saline solution was randomly administered in both hindlimbs. Treatment was repeated once a week. Animal groups were euthanized at 1 (T1), 2 (T2) or 4 (T4) weeks. Histological assessment showed that PRGF intralesional injection induced a significant decrease of inflammatory cells density, significant higher centrally nucleated fibers percentage and significantly smaller fibrotic areas compared to Saline-treated muscles at T1, T2 and T4. Also, lower vascular density, with lower capillaries cross-sectional area, in PRGF group compared to Saline was observed. Biochemical analysis revealed a significant higher expression level of *MYOD1*, *MYF5* and *MYOG* genes in PRGF groups at T1 compared to Saline treated muscles. At ultrastructural level, PRGF groups presented scarce edema and loss of connective tissue structure, as well as higher mitochondrial density adequately associated to the sarcomere unit in contrast to the Saline group. In conclusion, histological, biochemical, and ultrastructural results showed that PRGF treatment improved muscle regeneration process leading to more mature histological aspect in newly formed muscle tissue after a surgically induced grade II muscle injury.

**Supplementary Information:**

The online version contains supplementary material available at 10.1186/s12917-022-03491-2.

## Background

Muscle injuries are common lesion in athletes and workers that causes pain and disability with significant social impact and economic losses [1–3]. Grade II muscle injury is a prevalent lesion registered in sport medicine, comprising over 35% of all sports-related injuries, with a high incidence of lesion recurrence after returning to activity [1–4].

An inflammatory response occurs when muscle fibers are partially severed, and different inflammatory cells are sequentially attracted to the damaged area [5, 6]. This cascade of events facilitates myogenesis via phagocytosis of cellular debris, and release of chemoattractants and growth factors toward the injured muscle [7]. Modulation of the inflammatory response plays a crucial role to achieve a successful muscle healing process [8, 9]. Furthermore, after muscle rupture an angiogenic stimuli is activated in the injured area with the intention of providing the newly formed tissue with oxygen, other nutrients, and blood-derived cells, as well as removing waste-metabolic products [10].

Skeletal muscle tissue presents reparation capacity led by an intrinsic mechanism conducted by a group of undifferentiated reserve cells called satellite cells [10]. In response to injury, these cells differentiate into myoblasts, and form multinucleated myotubes. The newly formed myotubes then fuse with part of the healthy muscle stump [11]. Regenerating myofibers are characterized as centrally nucleated fibers (CNF), so they are considered criteria for muscle regeneration [10, 12, 13]. Likewise, MYOD1, Myf5 and Myogenin (MYOG) are muscle regulatory factors that are up-regulated when satellite cells are activated to proliferate in skeletal muscle repair [12].

The cellular events described during muscle reparation are orchestrated by a pool of growth factors and cytokines, most of which are released by platelets [2, 3, 10]. Platelet rich plasma (PRP) therapies have been used to treat acute muscle injuries in both experimental and clinical scenario [14–19]. PRP therapy induces an angiogenic stimulus and these new capillaries allow the transport of oxygen and nutrients to the healing tissue [20]. Furthermore, plasma rich in growth factors (PRGF), a particular type of autologous PRP that contains a moderate platelet concentration (2–3 fold above peripheral blood) with absence of leukocytes and erythrocytes [21], has been successfully proposed to improve muscle healing in clinical practice [2, 22]. Some clinical and experimental results reported after using PRP to manage muscle injuries are quite variable and not conclusive [3, 17].

The purpose of this experimental study is to investigate muscle regeneration properties of intralesional PRGF therapy for surgically induced grade II muscle injury in a sheep model. It was hypothesized that this treatment could modulate inflammation and enhance regeneration properties to restore normal or nearly skeletal muscle tissue.

## Methods

### Animals

Twenty-one skeletally mature 2- to 3-year-old female sheep, weighing 50–55 kg were included in the study. All sheep used in this study were deemed healthy beforehand and did not suffer from any orthopedic or systemic conditions. Animals were randomly divided into three groups, T1 (n = 7), T2 (n = 7), and T4 (n = 7), according to the time when they were sacrificed along the experiment, at 1 week, 2 weeks, or 4 weeks after treatment, respectively. All procedures were carried out following the guidelines of the European Union (Directive 2010/63/EU) and approved by the Bioethical Committee on Animal Research at Andalucía (Junta de Andalucía, reference 12/09/2018/138). In addition, authors affirm that the research was conducted in accordance with the ARRIVE guidelines.

### Surgical procedure

Sheep were anaesthetized and both thighs were aseptically prepared for surgery. First, a sonographer performed an ultrasound evaluation to confirm the absence of abnormalities in biceps femoris muscle (edema, structural disorganization and/or fibrosis). In order to standardize the exact point of the surgical lesion, the thickest muscle area to perform the grade II muscle injury was measured mid-point in between the greater trochanter and the proximal aspect of the lateral condyle of the femur. A surgical muscle laceration equivalent to grade IIc injury, consisting of a partial tears of muscle fibers, was performed under ultrasound guidance in the biceps femoris muscle involving 70% of the muscle fibers in all the sheep using a scalpel blade #22 (Aesculap AG, B/Braun, Tuttlingen, Germany) [23]. The magnitude of the grade II muscle rupture was confirmed by ultrasound according to the grading scale suggested by Hamid et al. [23] and Peetrons et al. [24]. Then the fascia lata, subcutaneous and skin layers were sutured in separated planes. The surgical procedure was completed on both thighs. All the animals were operated by the same surgeon. Finally, the wound was covered using sterile dressing. Antibiotic [amoxicillin-clavulanic acid (Synulox®, Zoetis, Madrid, Spain), 10 mg/kg IM] and analgesic [buprenorphine (Buprex® 0,3 mg, Schering-Plough, Madrid, Spain), 0.02 mg/kg/8 h IM] treatment were given for 3 and 5 days, respectively. No anti-inflammatory drug was used anytime. Animals were hospitalized in an indoor stable and were allowed to move freely without splinting.

### PRGF preparation

Blood was collected just prior to treatment from the jugular vein of each animal and was divided into four sterile tubes containing 3.8% trisodium citrate. Each 5-mL extraction tube contained 0.5 mL of anticoagulant. Blood was centrifuged at 630 g for 8 min according to the method reported to obtain PRGF (PRGF-Endoret®, BTI, Vitoria, Spain) in sheep [25]. The fraction considered PRGF was the deeper layer of the centrifuged plasma just over the buffy coat and was selected with the use of a pipette to avoid the aspiration of white and red cells. The PRGF fraction volume from each of the four tubes was combined to obtain a total volume of 2 ml per animal.

### PRGF treatment injection

Sheep were sedated for treatment injection 48 h after muscle injury. First, hematoma was evacuated from the muscle laceration site using a 21-G needle under ultrasound guidance. PRGF or Saline solution (NaCl 0,9%, BBraun medical, Barcelona, Spain) infiltrations were randomly assigned for each thigh. Platelets contained in PRGF (2 mL) were activated just prior to treatment by adding 50 µL of calcium chloride 10% for 1 mL of plasma ratio, and injected directly into the injured site under ultrasound guidance as previously described in Bubnov et al. [26]. The time delay between blood collection and PRGF application was less than 1 h. Saline solution (2 mL) was injected adding 50 µL of calcium chloride 10% for 1 mL of Saline ratio, under ultrasound guidance. PRGF or Saline solution infiltrations were repeated weekly under ultrasound guidance to complete, one more injection in T2 group, and two more infiltrations in T4 group. The researcher was blinded to the treatment applied on each limb.

### Duration of the study

All the animals were humanely sacrificed at 1 week (T1 group), 2 weeks (T2 group), or 4 weeks (T4 group) after the first treatment with PRGF or Saline solution, by using a standard protocol with T61™ (Intervet Schering Plough Animal Health; Salamanca, Spain).

### Histological study

After euthanasia, biceps femoris muscles of both thighs were dissected proximal-distally to the damaged area in each animal. Muscle damage area was divided, so one half was used for histochemical evaluation, and the other half was used for ultrastructural and biochemical studies. Samples of the vastus lateralis quadriceps femoris were taken and used as a normal skeletal muscle control (Control group). Samples were oriented keeping muscle fibers along their longitudinal axis.

#### Histochemical study

For histochemical examination, muscles samples were fixed in 10% neutral buffered formalin and embedded in paraffin. Four µm-thick sections were stained with Hematoxylin and Eosin (HE) and Masson Trichrome with aniline blue (MT) (Bio-Optica, Milan, Italy). Histological images were evaluated using a photomicroscope (Axiophot; Carl-Zeiss, Oberkochen, Germany) with an attached digital camera controller (DS-L1 camera control unit, Nikon).

From HE and MT stained sections, five histological images per slide were randomly taken at 200x magnification and were analyzed with ImageJ software (Java-based image processing program, Oracle corporation, Austin, Texas, USA). HE stained histological images were used to study the inflammatory cells density (cells/mm^2^), blood vessels density (blood vessels/mm^2^), blood vessels area (µm^2^) and percentage of centrally nucleated fibers with respect to total muscle fibers. MT stained histological images were used to assess areas of fibrillar collagen to estimate the percentage of fibrotic areas (FA) development.

#### Transmission electron microscopy study

For transmission electron microscopy (TEM) evaluation, all the muscle samples were fixed in glutaraldehyde 2% for 48 h at 4 ºC and included in Araldite. Ultrathin sections were stained with uranyl acetate and plumb citrate and evaluated using a transmission electronic microscope Jeol-1400 (JEOL Ltd., Hertfordshire, UK).

### Biochemical study

Muscle tissue samples stored at -80 ºC were thawed and processed for RNA extraction with TRI reagent (Sigma-Aldrich CO, St Louis, MO, USA). Total RNA was quantified by spectrophotometry (ND-1000, Nanodrop Technologies, Wilmington, DE, USA). Afterwards, total RNA treated with DNAse was used to analyze mRNA expression of MYOD1, MYF5, MYOG and Myostatin (MSTN) genes by RT-PCR using SensiFAST SYBR No-ROX One-Step kit (Bioline Reagents Limited, UK) according to the manufacturer instructions. RT-PCR was performed using a Lightcycler 480 equipment (Roche Molecular Systems, Inc., Indianapolis, IN, USA). Primer sequences are shown in Table 1. The gene expression was calculated following the 2^−ΔΔ Ct^ method using *GAPDH* as housekeeping gene.

**Table 1:** Primer sequences used for RT-Q-PCR.

Genes	Forward	Reverse
***MYOD1***	5’-GCA ATC CGC TAT ATC GAA GG-3’	5’-GTA AGC GCG GTC GTA GC-3’
***Myf5***	5’-AAG GTG GAG ATC CTC AGG AA-3’	5’-ATT CAG GCA TGC CAT CAG AGC AAC-3’
***MYOG***	5’-CCG TGG GCG TGT AAG GTG TG-3’	5’-CCT CTG GTT GGG GTT GAG CAG-3’
***MSTN***	5’-CCAGGA GAA GAA GGA CTG AATC-3’	5’-AAA AAT TCA CAT TCT CCA GAG CAG T-3’
***GAPDH***	5’-CCT GGA GAA ACC TGC CAA GT-3’	5’-GCC CAA TTC ATT GTC GTA CCA-3’

### Statistical analysis

The data were statistically evaluated using SPSS 17.0 software (SPSS Inc, Chicago, USA). All variables were analyzed for normality of distribution by the Kolmogorov-Smirnov test. Histochemical variables (inflammatory cells density, blood vessels density, blood vessels area, percentage of CNF, and percentage of FA), and level of expression of MYOD1, MYF5, MYOG and MSTN were expressed as mean ± standard deviation. A mixed general lineal model was performed to analyze each variable with respect to the muscles sample (PRGF group, Saline solution group or Control group). Furthermore, each variable was analyzed with respect to different timepoints (T1, T2 and T4) throughout the study. A one-way ANOVA with post hoc Bonferroni test were used to evaluate differences between groups (Control group vs. PRGF or Saline groups; PRGF group vs. Saline group). Significance level was standardized as p < 0.05.

## Results

All animals reached the end of the study without incidents or adverse effects.

### Histochemical study

**Fig. 1:**
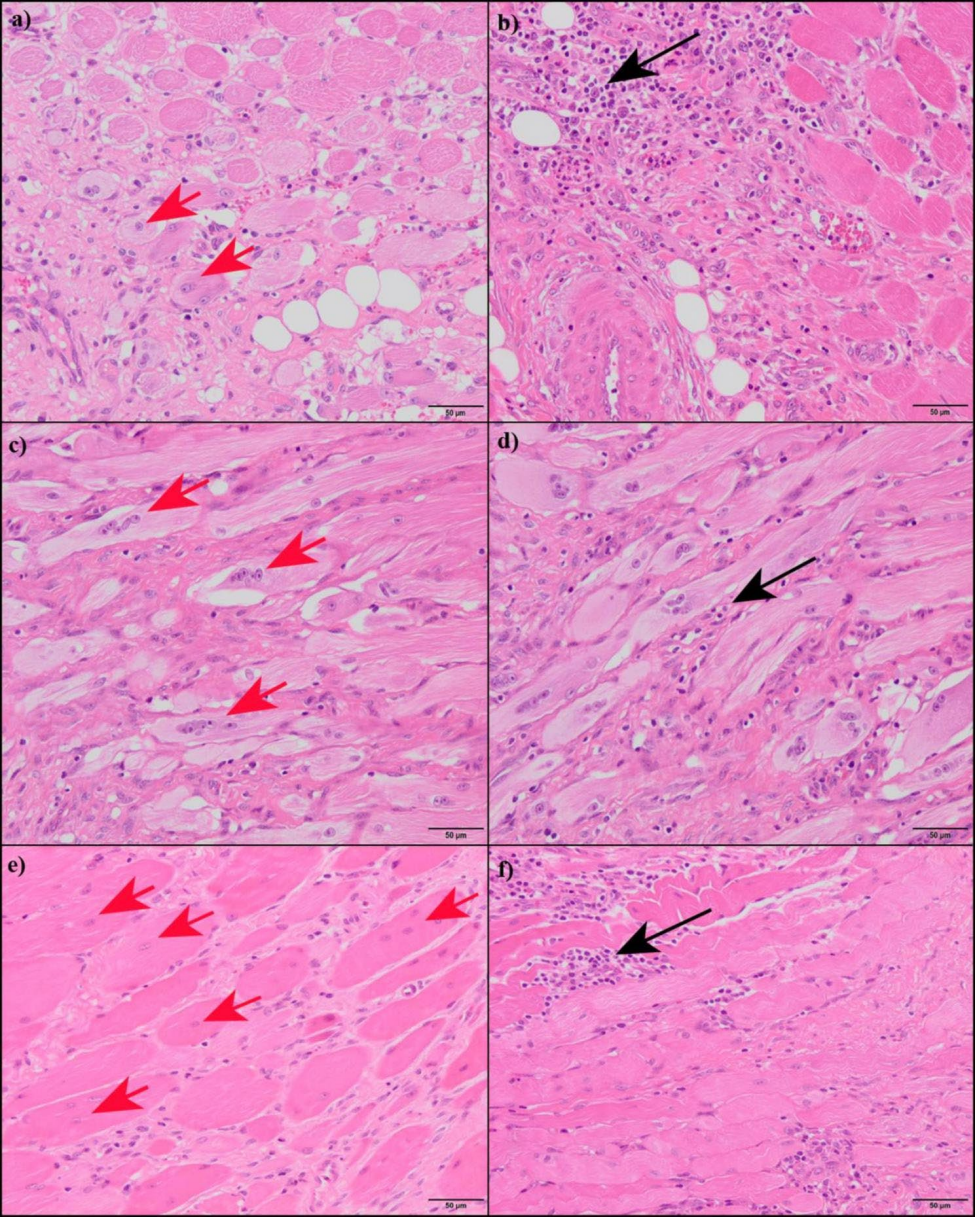
Representative histological sections stained with HE from grade II muscle injury in sheep, treated with PRGF (a, c, e) or Saline solution (b, d, f); at 1 (a, b), 2 (c, d) and 4 (e, f) weeks after the surgical muscle damage. Black arrows point at inflammatory cells infiltration; red arrows point at Centrally Nucleated Fibers (CNF). Muscles treated with PRGF showed statistically significant less inflammatory cells infiltration, and higher percentage of CNF than those muscles injected with Saline solution along the study

PRGF group, Saline solution group and Control group results are presented in Table 2. Normal skeletal muscle showed absence of inflammatory cells infiltration. Inflammatory cells density increased significantly in both groups treated with PRGF or Saline solution after the surgical injury. Damaged muscles treated with Saline solution showed significant higher inflammatory cells density compared to PRGF-treated group (Fig. 1). This difference was maintained at T1, T2 and T4 (p = 0.001).

**Table 2:** Histochemical study parameters results for PRGF group, Saline solution group and Control group (normal skeletal muscle) at 1 (T1), 2 (T2) and 4 (T4) weeks after treatment

Variable	Group	Group	Value (mean ± SD)
Inflammatory cells density (cells/mm^2^)	T1	PRGF	47 ± 13^(+,*)^
		Saline solution	87 ± 18^(+,*)^
		Control	0 ± 0
	T2	PRGF	52 ± 16^(+,*)^
		Saline solution	101 ± 40^(+,*)^
		Control	0 ± 0
	T4	PRGF	59 ± 16^(+,*)^
		Saline solution	107 ± 24^(+,*)^
		Control	0 ± 0
Vascular area (µm^2^)	T1	PRGF	303 ± 77^(+,*)^
		Saline solution	497 ± 127^(+,*)^
		Control	40 ± 1
	T2	PRGF	173 ± 25^(+,*)^
		Saline solution	373 ± 105^(+,*)^
		Control	40 ± 2
	T4	PRGF	160 ± 70^(+)^
		Saline solution	224 ± 96^(+)^
		Control	39 ± 2
Vascular density (blood vessels/mm^2^)	T1	PRGF	21 ± 6^(+)^
		Saline solution	26 ± 6^(+)^
		Control	12 ± 2
	T2	PRGF	16 ± 4^(+)^
		Saline solution	20 ± 4^(+)^
		Control	10 ± 3
	T4	PRGF	14 ± 3^(+,*)^
		Saline solution	20 ± 3^(+,*)^
		Control	10 ± 2
Centrally nucleated fibers (%)	T1	PRGF	27 ± 5^(+,*)^
		Saline solution	16 ± 5^(+,*)^
		Control	0 ± 0
	T2	PRGF	36 ± 11^(+,*)^
		Saline solution	25 ± 5^(+,*)^
		Control	0 ± 0
	T4	PRGF	50 ± 10^(+,*)^
		Saline solution	34 ± 6^(+,*)^
		Control	0 ± 0
Fibrotic areas (%)	T1	PRGF	24 ± 3^(+,*)^
		Saline solution	32 ± 5^(+,*)^
		Control	0 ± 0
	T2	PRGF	31 ± 5^(+,*)^
		Saline solution	42 ± 4^(+,*)^
		Control	0 ± 0
	T4	PRGF	22 ± 3^(+,*)^
		Saline solution	34 ± 5^(+,*)^
		Control	0 ± 0

^(+)^Statistically significant difference with respect to Control group (p < 0.05)

^(*)^Statistically significant difference between PRGF and Saline solution groups (p < 0.05)

Regarding vascularization analysis, Control group showed small capillaries distributed through the muscular tissue with low vascular density. After surgical grade II injury of biceps femoris, both PRGF-treated muscles and those injected with Saline solution suffered an important increase in the vascular area, showing significantly larger size capillaries than normal skeletal muscle. PRGF treated muscles exhibited statistically significant smaller vascular area than in those treated with Saline solution at T1 (p = 0.023) and T2 (p = 0.001). Vascular density was significantly increased in both surgically injured muscles injected with PRGF or Saline solution compared with the intact skeletal muscle. All the muscles showed a significant progressive drop in vascular density along the study. PRGF groups exhibited lower vascular density than Saline solution groups with significant differences at T4 (p = 0.019)

CNF were not observed in normal skeletal muscle. After the surgical damage of biceps femoris, a significant progressive increase of the percentage of CNF was registered from T1 to T4 groups in PRGF and Saline solution groups. Comparison between PRGF and Saline treatments revealed that percentage of CNF was significantly higher in muscles injected with PRGF than those treated with Saline solution at T1, T2 and T4. This difference was maintained throughout the experiment (p = 0.001) (Fig. 1)

**Fig. 2:**
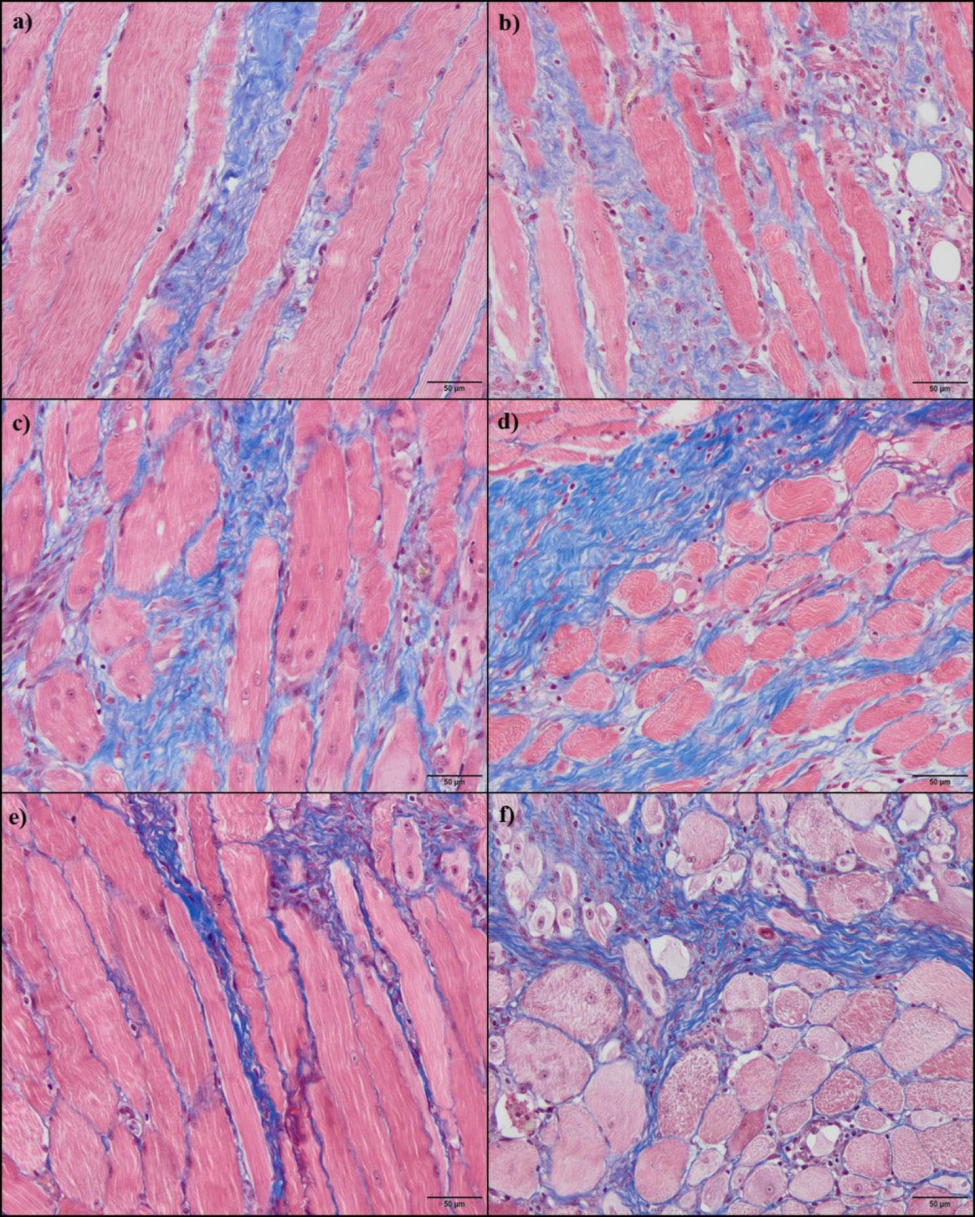
Representative histological sections stained with MT from grade II muscle injury in sheep, treated with PRGF (a, c, e) or Saline solution (b, d, f); at 1 (a, b), 2 (c, d) and 4 (e, f) weeks after the surgical muscle damage. Statistically significant higher fibrotic areas (blue-stained) were showed in muscles treated with Saline solution than in PRGF-treated muscles

Histological study showed absence of fibrotic areas in the Control group. During healing process, a significant increase in percentage of FA with respect to the total area were registered in PRGF or Saline solution groups at T1 and T2. Subsequently, this parameter decreased at T4 for both groups. Significantly higher fibrotic areas were described in muscles treated with Saline solution, in comparison with those muscles infiltrated with PRGF. This difference was maintained along the study from T1 to T4 groups (p = 0.002) (Fig. 2).

### Transmission Electron microscopy study

**Fig. 3:**
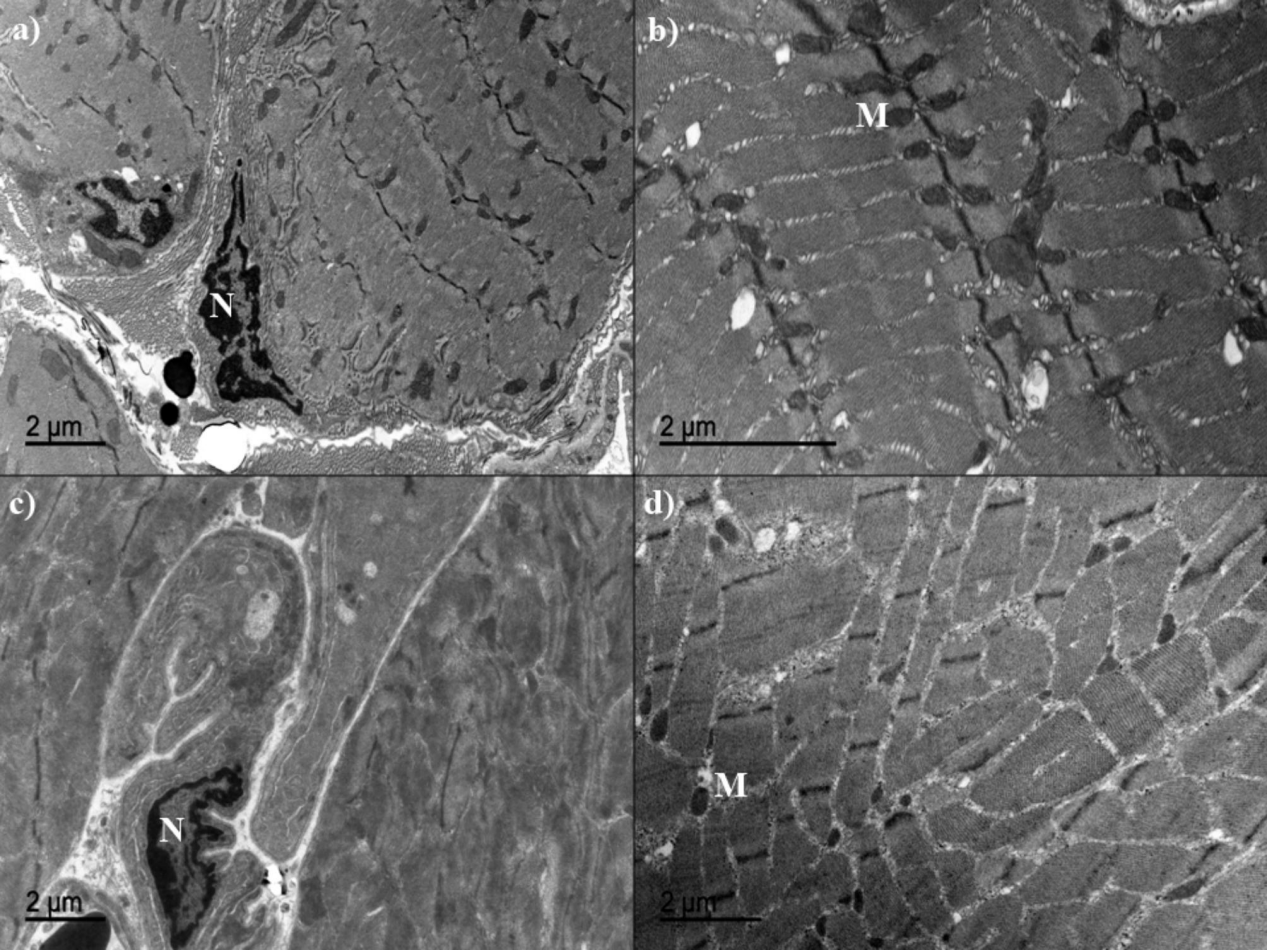
Transmission electronic microscope photographs from grade II muscle injury in sheep treated with PRGF (a, b), and Saline solution (c, d); at four weeks after the surgical damage. N: muscle fiber nucleus; M: mitochondria

Normal skeletal muscle showed typical sarcomere structure organized parallel to each other and distributed along the muscle fiber. Injured muscular tissue presented morphology compatible with hypertrophic phenomena, with differences depending on the therapy received throughout the experiment (Fig. 3). At T1 and T2, PRGF treated muscles showed scarce edema and loss of connective tissue structure. In contrast, Saline groups presented diffuse edema with T-tube and endoplasmic reticulum dilated, as well as disorganization in the connective tissue structure. At T4, PRGF-treated muscles exhibited physiologic hypertrophic changes with moderate undulating fibrils, and high mitochondrial density associated to the “Z” band of the sarcomere (Fig. 3a and b). However, Saline-treated muscles revealed pathologic hypertrophic changes with muscle fibrils growing uncoordinatedly non-parallel to the muscle fiber axis, as well as low mitochondrial density randomly distributed along the muscular tissue and many times not associated to the sarcomere (Fig. 3c and d).

### Biochemical study

Level of expression of myogenic genes *MYOD1*, *MYF5* and *MYOG*, as well as the inhibitor gene *MSTN*, from intact skeletal muscle and injured muscles treated with PRGF and Saline solution are described in Table 3. *MYOD1*, *MYF5* and *MYOG* genes showed significantly enhanced level of expression in the damaged muscles with respect to the Control group along the experiment. At T1, muscles treated with PRGF showed significantly higher level of expression of *MYOD1*, *MYF5* and *MYOG*, in comparison with the group of muscles injected with Saline solution (p = 0.025; p = 0.03; p = 0.025, respectively). However, on later stage of muscle healing at weeks 2 and 4, no differences were registered between the PRGF and Saline groups. Regarding *MSTN*, as inhibitor gene of muscle regeneration, no statistical differences were registered in the comparison either between injured muscles and Control group, or between PRGF and Saline groups throughout the experiment.

**Table 3:** Level of expression of MYOD1, MYF5, MYOG and MSTN genes for PRGF group, Saline solution group and Control group (normal skeletal muscle)at 1 (T1), 2 (T2) and 4 (T4) weeks after treatment

Variable	Group	Treatment	Value (mean ± SD)
MYOD1	T1	PRGF	22,97 ± 2,86^(+,*)^
		Saline solution	2,52 ± 0,27^(+,*)^
		Control	0,90 ± 0,09
	T2	PRGF	28,67 ± 5,76^(+)^
		Saline solution	26,39 ± 3,19^(+)^
		Control	0,90 ± 0,10
	T4	PRGF	45,25 ± 6,41^(+)^
		Saline solution	40,5 ± 5,29^(+)^
		Control	0,90 ± 0,10
MYF5	T1	PRGF	24,80 ± 4,52^(+,*)^
		Saline solution	9,53 ± 1,08^(+,*)^
		Control	0,78 ± 0,08
	T2	PRGF	74,13 ± 10,38^(+)^
		Saline solution	78,36 ± 8,43^(+)^
		Control	0,78 ± 0,08
	T4	PRGF	81,69 ± 9,69^(+)^
		Saline solution	81,55 ± 7,54^(+)^
		Control	0,78 ± 0,09
MYOG	T1	PRGF	23,43 ± 3,94^(+,*)^
		Saline solution	8,36 ± 1,91^(+,*)^
		Control	0,83 ± 0,08
	T2	PRGF	31,87 ± 3,93^(+)^
		Saline solution	25,18 ± 3,79^(+)^
		Control	0,85 ± 0,08
	T4	PRGF	40,62 ± 8,16^(+)^
		Saline solution	31,87 ± 3,46^(+)^
		Control	0.85 ± 0,08
MSTN	T1	PRGF	0,22 ± 0,03
		Saline solution	0,22 ± 0,04
		Control	0,76 ± 0,06
	T2	PRGF	0,53 ± 0,10
		Saline solution	0,81 ± 0,07
		Control	0,76 ± 0,06
	T4	PRGF	0,75 ± 0,08
		Saline solution	0,59 ± 0,11
		Control	0,76 ± 0,06

^(+)^Statistically significant difference with respect to Control group (p < 0.05). ^(*)^Statistically significant difference between PRGF and Saline solution groups (p < 0.05)

## Discussion

Muscle injuries are one of most frequently reported lesions related to the sports world [3]. Acceleration of the muscle regeneration with no appearance of recurrences is the main objective of an effective therapeutic strategy [10]. On this line, platelet-rich therapies have been demonstrated to enhance the healing of musculoskeletal injuries through the growth factors and cytokines pool released from platelets and plasma [27].

PRGF is an autologous treatment that accelerates tissue regeneration and reduces the recovery period, improving the quality of tissue repaired with the aim of decreasing relapse incidence [28–30]. There are few publications with the use of PRGF in the treatment of grade II muscle injuries [2, 22]. To our knowledge, PRGF has not been employed in any experimental setting to evaluate its effect on muscle regeneration after a surgical grade II injury on the biceps femoris in a sheep model.

In the present study, the anatomical references considered and ultrasound guidance used were useful to locate the thickest muscle area to precisely determine the surgical lesions site. Furthermore, the grade II muscle injuries in the biceps femoris muscle was accurately standardized using an ultrasound muscle injury grading scale previously reported [23]. Ultrasonography was used to visualize the scalpel blade during the procedure, and also to assess that a grade IIc muscle injury had been performed after surgery. Subsequently, according to these criteria an accurate, homogeneous, and repeatable sheep model of grade II biceps femoris muscle injury was developed to evaluate the effect of PRGF therapy on muscle regeneration. In the present study, a tear of 70% of muscle fibers was performed in order to create a demanding muscle damage to assess PRGF therapy in comparison with the saline group and a control muscle. Similarly, PRP treatment with rehabilitation program has been successfully utilized to manage similar size of muscle tears in people [23].

When muscle fibers are partially severed, inflammatory phenomena occurs and different inflammatory cells are sequentially attracted to the damaged area [5, 6]. Several growth factors contained in PRP contribute to the modulation and subsequent resolution of inflammation after muscle tears [2, 31]. In this study, PRGF groups showed a significant decrease in inflammatory cells density compared with Saline-treated muscles in this study. This finding suggested an anti-inflammatory effect of PRGF on muscle repair since the first week of the regeneration process. However, Borrione et al. [32] described an early increase in inflammatory cells infiltration in muscles treated with PRP in contrast to the untreated group, after a surgically created grade II muscle injury at days 2 and 5. In addition, Delos et al. [15] registered no differences between PRP and Saline treatments in inflammatory cells density after muscle contusion at days 1, 4, 7, 10, and 14. Both experimental studies [15, 32] used a platelet concentration in PRP of at least 4-fold above peripheral blood. Although, excessive platelet concentration in the PRP could lead to non-desired effect in tissue regeneration process [33–35]. Furthermore, Delos et al. [15] included the leukocytes layer in their PRP. Therefore, leukocytes could interfere with the beneficial effects that some growth factors have on modulating the inflammatory response due to alteration of the growth factor release profile, as well as contributing to the increase of proinflammatory cytokines [6, 36, 37]. The anti-inflammatory effect of PRGF has been previously reported in other tissues [27, 28, 38].

The restoration of vascular supply is prerequisite for subsequent morphological and functional recovery of the injured muscle [10]. In the present study, smaller size capillaries and lower vascular density were observed in PRGF-treated muscles in comparison with Saline group updating that PRGF therapy modulated neovascularization phenomena. However, Borrione et al. [32] described no significant effect of PRP on blood vessels density and blood vessels diameter in damaged muscles with respect to the untreated group.

Muscle regeneration have been evaluated in several experiments using CNF counting as histological criteria [13, 15]. Our results showed that PRGF treatment induced higher muscle regeneration stimulus after a grade II muscle injury due to a significant higher percentage of CNF than Saline-infiltrated muscles. Nevertheless, Delos et al. using a PRP that included platelet concentration superior to 4 times the whole blood level reported no effect of the therapy on muscle reparation [15]. Therefore, moderate platelet concentration included in PRGF could enhance the muscle regeneration.

During muscle repair process, connective tissue occupies the gap created in between the ruptured myofibers [10]. The production of connective tissue and subsequent scar formation at the site of muscle injury is considered one of the most important pathological steps in muscle healing, and therefore it is extremely associated to relapses [10, 39]. Thus, with the aim to reduce the fibrotic scar, novel therapies are needed to deal with grade II muscle injuries. In the present study, after surgical grade II muscle injury, MT staining revealed significantly smaller fibrotic areas in the group of muscles treated with PRGF with respect to Saline-treated muscles. In contrast, Delos et al. [15] and Cunha et al. [40], reported that damaged muscles injected with PRP showed no difference in fibrotic areas compared to control group. These authors used a PRP that contained a platelet concentration superior to 4 times above peripheral blood, higher than the PRGF platelet concentration (2–3 folds) utilized in our study. TGF-β1 is the growth factor that initiates the production of fibrosis-related proteins, as well as down-regulating the expression of myogenic proteins [41, 42]. Several studies have demonstrated that fibrotic tissue in regenerated muscle could be prevented using either a TGF-β1 neutralization antibody or an antifibrotic agent, thereby improving the recovery of strength in the injured muscle, as well as promoting muscle regeneration [13, 34, 43]. Consequently, in contrast to our experiment, excessive TGF-β1 may disguise the positive effect that other growth factors have on muscle fibrosis and regeneration.

The hypertrophic reaction described in all the surgically damaged muscles is associated to the activation of satellite cells and the beginning of its division, with the aim of regenerating the injured muscle [44]. The ultra-structural evaluation in this study revealed that during the first 2 weeks PRGF therapy induced better connective tissue structure and less edema than Saline group. Therefore, these findings are correlated with minor inflammatory cells infiltration described in the HE stained sections. Besides that, at week 4 of study, PRGF was associated with less undulated muscle fibrils, which was indicative of physiologic and controlled regeneration process [44]. Moreover, PRGF treated muscles group showed higher mitochondrial density located more efficiently attached to the “Z” band of the sarcomere. Mitochondrial organelles are in charge of oxidative metabolism, which constitutes the principal energy pathway in the mature skeletal myofibers [10, 45]. It may suggest that muscles injected with PRGF presented higher metabolism rate to accelerate the regeneration process in comparison with those injected with Saline solution.

At biochemical level, the expression of muscle regulatory factors becomes objective criteria that have been utilized to evaluate muscle regeneration [16, 18]. In concordance with previous studies [16, 18], our results showed that PRGF-treated muscles described statistically higher level of expression of *MYOD1*, *MYF5* and *MYOG* in the precocious stage of muscle regeneration at one week after grade II muscle rupture. This finding, together with statistically higher percentage of CNF previously described in the group of muscles treated with PRGF, suggests that PRGF therapy enhances the regeneration stimulus of muscle, accelerating the repair process, and subsequently it may shorten the convalescence period after grade II muscle injury. On the other hand, *MSTN* level of expression did not show statistical differences with respect to the PRGF of Saline solution therapy along the study. Thereby, muscle regeneration process is not being inhibited despite PRGF o Saline solution injection.

Several types of PRP have been used in clinical setting with the goal of accelerating the muscle regeneration process, as well as avoiding fibrotic tissue scar at the end of muscle healing procedure [17, 19, 22, 26, 46–52]. Protocols to obtain PRP, its final composition, and the method to assess its clinical effect on muscle regeneration are different from one study to the other. Subsequently, clinical results with the use of PRP, as general concept of platelet rich concentrate, are extremely variable and non-consistent in grade II muscle injuries. Hamilton et al. [17] evaluated the efficacy of PRP application in reducing time to return to sport after a grade II muscle injury in athletes. They recorded no benefits of a single dose of PRP injection over an intensive rehabilitation program with MRI evaluation [17]. Martinez-Zapata et al. [50] reported that a single dose of PRP injection did not significantly improve the time to completely heal a grade II muscle rupture compared to the control group. Both clinical studies used a PRP which contained 5–9 fold platelet concentration above peripheral blood [17, 50]. Besides that, Hamilton et al. [17] included 5-fold leukocytes concentration above peripheral blood in the PRP composition. Excessive platelet concentration and consequently unbalanced concentrations of certain growth factors such as TGF-β1 are associated to pathologically newly formed muscle tissue with the presence of fibrotic scar [13, 34, 43]. Moreover, leukocytes incorporated to the PRP composition are rich in metalloproteases that destroy growth factors and promote inflammatory phenomena [6, 37]. In addition, a single dose of PRP was injected in a grade II muscle injury in both clinical studies [17, 50]. However, 3 weekly infiltrations were performed in our experimental model. Thus, it would reproduce a moderate supraphysiological stimulus maintained throughout the muscle regeneration. Likewise, PRGF has been successfully used in clinical setting [2, 22]. Loo et al.[22] described 3 weekly intralesional injections of PRGF under ultrasound guidance for adductor longus tear treatment. The patient reported good pain relief, likely associated with reduced inflammation, and was able to get back to competition in 4 weeks [22]. The absence of leukocytes, and moderate platelet concentration described in the PRGF may induce a balanced stimulus on the muscle regeneration. In consequence, PRGF could accelerate the muscle regeneration process and enhance the quality of newly formed muscle tissue, with the presence of less fibrotic scar after a grade II muscle rupture in clinical setting.

There are several limitations to this study. First, ultrasonographic follow-up was not evaluated to check the muscle repair stage on ultrasound-imaging. The goal of the present study was to evaluate the histological and genomic effect of PRGF on muscle regeneration process. Therefore, ultrasound technique was only used as guidance to intralesional infiltration of PRGF or Saline solution, as previously described [26]. Second, functional testing was not performed. Since ovine specie is a prey in the wild animal world, they do not show any pain in weight bearing unless the muscular damage is severe. In this line, visual analogue scale or similar would not provide adequate results on the pain the animal was experiencing. Nevertheless, electromyography is a standardized test that would have addressed objective measurements about the functionality of the damaged muscle [53]. We will strongly consider this technique for future study to elucidate the effect of PRGF on the time to return to total functionality in a grade II muscle tear. Third, a different skeletal muscle, vastus lateralis quadriceps femoris, was used as control muscle in the histological analysis. However, an identical histological aspect was found compared to biceps femoris and accordingly, for ethical reasons we decided not to include more animals in the study.

In conclusion, PRGF intralesional treatment produced a significant decrease in the inflammatory cell density, with lower vascular density and blood vessel area, in comparison with the Saline-treated muscles. PRGF stimulated a significant higher percentage of CNF, and also the expression of myogenic genes (*MYOD1*, *MYF5* and *MYOG*) was significantly higher in PRGF-treated muscles at the early stage of muscle regeneration. Furthermore, PRGF treatment leaded to newly formed muscle tissue with significant smaller fibrotic areas along the study, in contrast with Saline-treated muscles. These findings suggested an anti-inflammatory effect of PRGF on muscle repair, with an enhanced muscle regeneration stimulus, and improved repair quality after a surgically induced grade II muscle injury.

## Electronic supplementary material

Below is the link to the electronic supplementary material.

Supplementary Material 1

## Data Availability

All data are provided in the main manuscript file and supplementary material along with raw data file in respective section.
